# SUIHTER: a new mathematical model for COVID-19. Application to the analysis of the second epidemic outbreak in Italy

**DOI:** 10.1098/rspa.2021.0027

**Published:** 2021-09

**Authors:** N. Parolini, L. Dede’, P. F. Antonietti, G. Ardenghi, A. Manzoni, E. Miglio, A. Pugliese, M. Verani, A. Quarteroni

**Affiliations:** ^1^ MOX, Department of Mathematics, Politecnico di Milano, Milano, Italy; ^2^ Department of Mathematics, University of Trento, Trento, Italy; ^3^ Institute of Mathematics, École Polytechnique Fédérale de Lausanne (EPFL), Lausanne, Switzerland

**Keywords:** mathematical model, COVID-19, epidemic outbreak, parameter calibration, forecast analysis

## Abstract

The COVID-19 epidemic is the latest in a long list of pandemics that have affected humankind in the last century. In this paper, we propose a novel mathematical epidemiological model named SUIHTER from the names of the seven compartments that it comprises: susceptible uninfected individuals (*S*), undetected (both asymptomatic and symptomatic) infected (*U*), isolated infected (*I*), hospitalized (*H*), threatened (*T*), extinct (*E*) and recovered (*R*). A suitable parameter calibration that is based on the combined use of the least-squares method and the Markov chain Monte Carlo method is proposed with the aim of reproducing the past history of the epidemic in Italy, which surfaced in late February and is still ongoing to date, and of validating SUIHTER in terms of its predicting capabilities. A distinctive feature of the new model is that it allows a one-to-one calibration strategy between the model compartments and the data that are made available daily by the Italian Civil Protection Department. The new model is then applied to the analysis of the Italian epidemic with emphasis on the second outbreak, which emerged in autumn 2020. In particular, we show that the epidemiological model SUIHTER can be suitably used in a predictive manner to perform scenario analysis at a national level.

## 1. Introduction

The coronavirus disease 2019 (COVID-19) pandemic is a tremendous threat to global health. Since the first outbreak in early December 2019 in China, more than 1 834 573 deaths have been registered worldwide, while the estimated total number of confirmed cases is 84 511 153 up to 2 January 2021 [1]. The real number of people infected is unknown, but it is probably much higher. In this scenario, predicting the trend of the epidemic is of paramount importance to mitigate the pressure on health systems and to activate control strategies (e.g. quarantines, lockdowns and suspension of travel) aimed at containing the disease and delaying the spread.

As these predictions have vital consequences for the different actions taken by governments to limit and control the COVID-19 pandemic, the recent period has seen considerable growth of epidemiological mathematical models (e.g. [2–7]). However, estimates and scenarios emerging from modelling highly depend on different factors, ranging from epidemiological assumptions to, perhaps most importantly, the completeness and quality of the data on which models are calibrated. Since the beginning of the COVID-19 emergency, the quality of data on infections, deaths, tests and other factors has been spoiled by under-detection or inconsistent detection of cases, reporting delays and poor documentation. This has affected, and is still to date hampering, the intrinsic predictive capability of mathematical models.

Despite the lack or incompleteness of the available data, which makes modelling the current COVID-19 outbreak challenging, mathematical models are still vital to establish predictions within reasonable ranges, and can be adapted to incorporate the effects of public health authority interventions in order to estimate in advance their effectiveness and their impact on the spread of COVID-19. Building upon the well-known susceptible–infectious–recovered (SIR) model proposed in 1927 by Kermack & McKendrick [8], several generalizations have been formulated over the years by enriching the number of compartments, e.g. susceptible–exposed–infectious–recovered (SEIR), susceptible–infectious–susceptible (SIS), susceptible–exposed–infected–recovered–deceased (SEIRD), susceptible–exposed–infectious–asymptomatic–recovered (SEIAR), susceptible–infectious–recovered–susceptible (SIRS), susceptible–exposed–infectious–quarantined–recovered (SEIQR), maternally derived immunity–susceptible–exposed–infectious–recovered (MSEIR),…; we refer to, for example, [9–11] for an overview. Overall, these models have been abundantly applied to locally analyse COVID-19 outbreak dynamics in various countries (e.g. [6,12–14]).

However, the peculiar epidemiological traits of COVID-19 require models that are better able to accurately portray the mutable dynamic characteristics of the ongoing epidemic, with particular emphasis on two critical aspects: (i) the crucial role played by the undetected (both asymptomatic and symptomatic) individuals; (ii) the number of individuals who require intensive care unit (ICU) admission. This latter aspect is of paramount importance in designing realistic scenarios that incorporate the pressure of the epidemic on national health systems.

In this paper, we introduce a new mathematical model, named SUIHTER, based on the initials of the seven compartments that it comprises: susceptible uninfected individuals (*S*), undetected (both asymptomatic and symptomatic) infected (*U*), isolated infected (*I*), hospitalized (*H*), threatened (*T*), extinct (*E*), recovered (*R*). It is a system of coupled ordinary differential equations (ODEs) that are driven by a set of parameters that are indeed piecewise constant time-dependent functions. A first set of parameters denote the transmission rates due to contacts between susceptible and undetected, quarantined or hospitalized subjects. A second set of parameters mimics the rates at which *I* (isolated) and *H* (hospitalized) individuals develop clinically relevant or life-threatening symptoms. A further parameter indicates the probability rate of detection of previously undetected infected individuals. Another set of parameters indicates the rate of recovery for the four classes of infected subjects. Finally, the last parameters denote the mortality rates for the different compartments.

This SUIHTER model has been conceived to overcome some of the limitations that can be found in existing epidemiological models applied to the COVID-19 pandemic. On the one hand, some studies adopt simple SIR-like models [6,12,14], which have the advantage of a limited number of parameters to be calibrated but are unable to track the dynamics of different categories of infected individuals. On the other hand, other multi-compartmental models (e.g. [2,5]) have been proposed to account for the detailed knowledge of the clinical characterization for different classes of infected individuals according to the actual level of disease severity. However, it is not always possible (and, even when possible, it is not easy) to associate the multiple infected compartments with the available data. The SUIHTER model has been designed with the objective of creating the most compact model able to predict the different categories of infectious individuals that are considered relevant by the policymakers.

A key challenge in modelling the dynamics of the COVID-19 epidemic is represented by the large number of undetected cases. Indeed, the contribution to the spread of the epidemic by (often asymptomatic) undetected cases is too relevant to be neglected. Several authors [5,15,16] have attempted, using different strategies, to quantify the number of undetected infections and their effect on epidemic spread. In the present work, we propose a strategy for the initialization of those compartments that are not covered by the data (such as *susceptible*, *undetected* and *recovered*).

The model adopts a two-step calibration process based on a preliminary estimation of the model parameters that uses a least-squares (LS) minimization, followed by a Bayesian calibration performed through a Markov chain Monte Carlo (MCMC) algorithm.

The model has been adopted to simulate the second COVID-19 epidemic outbreak in Italy, began in autumn 2020 (and is still ongoing). In particular, we have investigated the capability of the model in forecasting the occurrence of a peak for the most relevant compartments with adequate advance notice. The results of the calibration, simulation by SUIHTER and predictions for Italy and the six largest Italian regions are also reported.

The outline of the paper is as follows: in §2, we introduce the SUIHTER mathematical model; §3 is devoted to the description of the calibration procedure; and §4 contains the numerical results along with the discussion. In §5, we draw our conclusions and we discuss some of the model’s limitations.

## 2. Mathematical model

**Figure 1.**
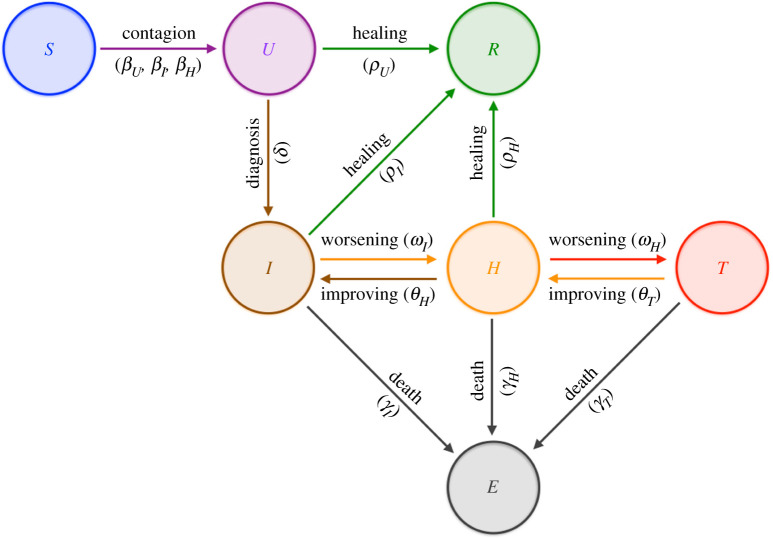
Interactions among compartments in SUIHTER model. (Online version in colour.)

The spread of COVID-19 has made it clear that it is of paramount importance to include in epidemiological models a compartment describing the dynamics of infected individuals who are still undetected. This is, for example, the case in [5]. However, some compartments presented in [5] (undetected asymptomatic infected and undetected symptomatic infected) are virtually impossible to validate since these classes of individuals cannot be traced in public databases (see [17]). For this reason, building upon [5], we propose a new model more suited to taking full advantage of publicly available data. In particular, our model is described by the following system of ODEs:

2.1 $$\begin{array}{rl} \dot{S}(t) & =-S(t)\frac{\beta_{U}U(t)+\beta_{I}I(t)+\beta_{H}H(t)}{N},\\ \dot{U}(t) & =S(t)\frac{\beta_{U}U(t)+\beta_{I}I(t)+\beta_{H}H(t)}{N}-(\delta +\rho_{U})U(t),\\ \dot{I}(t) & =\delta U(t)-(\rho_{I}+\omega_{I}+\gamma_{I})I(t)+\theta_{H}H(t),\\ \dot{H}(t) & =\omega_{I}I(t)-(\rho_{H}+\omega_{H}+\theta_{H}+\gamma_{H})H(t)+\theta_{T}T(t),\\ \dot{T}(t) & =\omega_{H}H(t)-(\theta_{T}+\gamma_{T})T(t),\\ \dot{E}(t) & =\gamma_{I}I(t)+\gamma_{H}H(t)+\gamma_{T}T(t),\\ \dot{R}(t) & =\rho_{U}U(t)+\rho_{I}I(t)+\rho_{H}H(t), \end{array}\}$$

where the compartments of the model are defined as follows (figure 1):

— S: number of *susceptible* (uninfected) individuals;
— U: number of *undetected* (both asymptomatic and symptomatic) infected individuals;
— I: number of infected individuals *isolated* at home;
— H: number of infected *hospitalized* individuals;
— T: number of infected *threatened* individuals being cared for in ICUs;
— E: number of *extinct* individuals;
— R: number of *recovered* individuals;

and N=S+U+I+H+T+E+R denotes the total population (assumed constant).

The model is characterized by the following 14 parameters, some of which are chosen as time-dependent piecewise polynomial functions:

— $\beta_{U}$, $\beta_{I}$, $\beta_{H}$ denote the transmission rates due to contacts between a susceptible subject and an undetected infected, a quarantined or a hospitalized subject, respectively;
— $\omega_{I}$ denotes the rate at which I-individuals develop clinically relevant symptoms, while $\omega_{H}$ denotes the rate at which H-individuals develop life-threatening symptoms;
— $\theta_{H}$ and $\theta_{T}$ denote the rates at which the health of H- and T-individuals improves and they return to the less critical I and H compartments, respectively;
— δ denotes the probability rate of detection, relative to undetected infected individuals;
— $\rho_{U}$, $\rho_{I}$ and $\rho_{H}$ denote the rate of recovery for three classes (U, I and H, respectively) of infected subjects;
— $\gamma_{I}$, $\gamma_{H}$ and $\gamma_{T}$ denote the mortality rates for the individuals isolated at home, hospitalized and being cared for in ICUs, respectively.

Since data available for the *recovered* cases do not include those individuals who recovered before being detected, we also propose a novel indicator that will be denoted as *recovered from detected* and that we define as

$$R_{D}(t)=\int_{t_{I}}^{t}(\rho_{I}I(\tau )+\rho_{H}H(\tau ))\;\text{d}\tau .$$

This indicator can be obtained in postprocessing from computed compartments and collects those individuals who recover after being detected.

In mathematical epidemiology, a fundamental quantity is the *basic reproduction number* (denoted by $R_{0}$), which is used to measure the transmission potential of a disease. It represents the average number of secondary infections produced by a typical case of an infection in a population where everyone is susceptible (see [9,11,18]). For our model, by using a similar argument to the one adopted in the proof of proposition 1 in [5], we find

2.2 $$R_{0}=\frac{\beta_{U}}{r_{1}}+\frac{\delta }{r_{1}}\left(\frac{\beta_{I}(r_{3}r_{4}-\theta_{T}\omega_{H})+\beta_{H}\omega_{I}r_{4}}{r_{2}r_{3}r_{4}-r_{4}\theta_{H}\omega_{I}-r_{2}\theta_{T}\omega_{H}}\right),$$

where $r_{1}=\delta +\rho_{U},r_{2}=\rho_{I}+\omega_{I}+\gamma_{I},r_{3}=\rho_{H}+\omega_{H}+\theta_{H}+\gamma_{H}$ and $r_{4}=\theta_{T}+\gamma_{T}$. For the sake of comparison (see eqn (32) in [5]), we observe that in the present context the characteristic polynomial q(s) of the Jacobian matrix associated with the linearization of (2.1) around the equilibrium configuration $\left(\bar{S},0,0,0,0,\bar{E},\bar{R}\right)$ with $\bar{S}+\bar{E}+\bar{R}=N$ is

$$q(s)=s^{3}p(s)\;\text{with}\;p(s)=D(s)-\bar{S}N(s),$$

where

$$D(s)=(s+r_{1})(s+r_{2})(s+r_{3})(s+r_{4})-(s+r_{1})\theta_{H}\omega_{I}-(s+r_{1})(s+r_{2})\theta_{T}\omega_{H}$$

and

$$\begin{array}{rl} N(s) & =(s+r_{4})\{\beta_{U}[(s+r_{2})(s+r_{3})-\omega_{I}\theta_{H}]+\beta_{I}\delta (s+r_{3})+\beta_{H}\delta \omega_{I}\}\\ & \;-\beta_{U}\omega_{H}\theta_{T}(s+r_{2})-\beta_{I}\delta \theta_{T}\omega_{H}. \end{array}$$

From a mathematical point of view, the reproduction number $R_{0}$ plays the role of a threshold value at the outset of the epidemic. If $R_{0}>1$, the disease spreads in the population; if $R_{0}<1$, the number of infected gradually declines to zero. Note that all factors in equation (2.2) are, as expected, actually positive. Furthermore, the expression (2.2) considerably simplifies upon assuming, as done in the remainder of this paper, that $\beta_{I}=\beta_{H}=\theta_{H}=\gamma_{H}=0$. Our SUIHTER model, as with other compartmental models, corresponds to a particular case of an integral model with arbitrary distribution of infectious time, for which $R_{0}$ is well known [18].

### (a) Model initialization

A critical issue is the way in which those compartments for which data are unavailable (*susceptible*, *undetected* and *recovered*) are initialized. In particular, when the analysis focuses on a late phase of the epidemic, as in the present investigation of the second epidemic outbreak in autumn 2020, it may be difficult to estimate those ‘initial’ values as a result of the simulation from day 0.

For these reasons, we have devised a strategy to estimate the number of *recovered* and *undetected* individuals based on the value of the *infection fatality ratio* (IFR), which is defined as the ratio between the number of deaths and the number of resolved cases (dead or recovered) at a specific time (ideally at the end of the epidemic)

2.3 $$\text{IFR}=\frac{E}{R+E}.$$

We assume that IFR will be roughly constant in time, at least over the first wave. By using the age-dependent estimates given in [19] the IFR can be estimated at around 1.2 % for Italy. The number of *recovered* individuals on a given day can then be computed from (2.3) based on IFR and the number of *expired* individuals. Moreover, the number of *undetected* individuals at a given time can be obtained by exploiting the detecting ratio at any given time as follows. We introduce a time-dependent *case fatality ratio* (CFR), which is defined as

2.4 $$\text{CFR}(t)=\frac{\Delta E(t)}{\Delta R_{D}(t)+\Delta E(t)},$$

where ΔE(t)=E(t+Δt/2)−E(t−Δt/2) and $\Delta R_{D}(t)=R_{D}(t+\Delta t/2)-R_{D}(t-\Delta t/2)$ denote the deaths and (detected) recovered cases observed in a time window of size Δt=28 days around a given time t. The number of *undetected* individuals at a given time is estimated by assuming that the detection ratio, that is, the percentage of detected cases with respect to the total number of positive cases, is computed as

2.5 $$\frac{I(t)+H(t)+T(t)}{U(t)+I(t)+H(t)+T(t)}\approx \frac{\Delta I(t)+\Delta H(t)+\Delta T(t)}{\Delta U(t)+\Delta I(t)+\Delta H(t)+\Delta T(t)}=\frac{\text{IFR}}{\text{CFR}(t+d)}.$$

Here, we have assumed that the variation in the number of total positive individuals in the time window [t−Δt,t+Δt] can be approximated by the variation in the resolved cases shifted by a confirmation-to-death delay d=13 (see [20]), namely

$$\Delta U(T)+\Delta I(t)+\Delta H(t)+\Delta T(t)\approx \Delta R(t+d)+\Delta E(t+d)=\frac{\Delta E(t+d)}{\text{IFR}}.$$

Similarly, we have assumed that the variation in the number of detected positive individuals in the time window [t−Δt,t+Δt] can be approximated by the variation in the resolved detected cases shifted by the same time delay

$$\Delta I(t)+\Delta H(t)+\Delta T(t)\approx \Delta R_{D}(t+d)+\Delta E(t+d)=\frac{\Delta E(t+d)}{\text{CFR}(t+d)}.$$

By using the available data for I, H, T and E, other than the value of S deduced under the assumption of constant total population and the estimate of CFR(t) given in equation (2.4), we estimate the initial conditions for R and U as

$$R(t)=\left(\frac{1}{\text{IFR}}-1\right)\;E(t)$$

and

$$U(t)=\left(\frac{\text{CFR}(t+d)}{\text{IFR}}-1\right)\;(I(t)+H(t)+T(t)),$$

from equations (2.3) and (2.5), respectively.

## 3. Parameter calibration

Model calibration through data fitting is essential to reproduce the past history of the epidemic and to perform short-term forecasts by inferring the epidemiological characteristics of COVID-19.

Here, we use data reported for isolated, hospitalized, threatened and extinct cases to estimate the parameters of the proposed SUIHTER model. In particular, we perform the calibration in two steps. Firstly, we find a set of parameter values using an (ordinary) LS estimator. Then, we perform a Bayesian calibration using an MCMC algorithm, starting from a prior distribution of the parameters centred about the LS estimate. Calibration of epidemiological models has already been performed in a Bayesian framework, following the pioneering paper by O’Neill & Roberts [21], for several infectious diseases [22–24]. In the case of the COVID-19 epidemic, Bayesian inference has been performed using simpler SIR [25,26], meta-community SEIR-like [2,4,13,27,28] and SEIAR [7] models, in the last case aiming at estimating nine parameters—including a dynamic, time-dependent contact rate β(t)—during the first outbreak of the COVID-19 epidemic. In addition to model calibration, our analysis also provides a numerical assessment of the predictive capability of the model, in forecasting with adequate advance notice the occurrence of a peak for the most relevant compartments.

System (2.1) can be recast in the following general form, which describes a system of ODEs for a state vector Y with $n_{e}$ components (or compartments): find $Y(t):[t_{I},t_{F}]\to R^{n_{e}}$ with $Y(t)={\left[Y_{1}(t),\ldots ,Y_{n_{e}}(t)\right]}^{T}$, such that

3.1 $${\text{Y}}^{\prime }(t)=\text{F}(t,\text{Y}(t);\text{p}(t))\;t\in (t_{I},t_{F}]$$

and

3.2 $$\text{Y}(t_{I})={\text{Y}}_{0},$$

where F is the right-hand side of system (2.1) and $Y_{0}\in R^{n_{e}}$ denotes the initial condition at time $t_{I}$ evaluated as discussed in §2a. The evolution of the system depends on $n_{\text{par}}$ time-dependent parameters, collected into the function $p(t):(t_{I},t_{F}]\to R^{n_{p}}$. Note that, at the discrete level, the nonlinear nature of system (2.1) is treated by considering an explicit (fourth-order Runge–Kutta) time discretization with time step Δt=1 day.

Let us partition the interval $I=[t_{I},t_{F}]$ into $n_{\text{ph}}$ phases, corresponding to different epidemic stages due to, for example, partial restrictions (such as lockdown measures) or different containment rules introduced by the government or by the local authorities, so that in each phase the environmental conditions may be considered fixed. Thus, in each phase, the values of the $n_{\text{par}}$ model parameters are assumed constant (but unknown), so that we can introduce the following set of admissible parameters:

3.3 $$P_{ad}=\{\text{p}(t):\text{p}(t){|}_{I_{k}}={\text{p}}_{k}\in [{\text{p}}_{L,k},{\text{p}}_{U,k}],\;k=1,\ldots ,n_{\text{ph}}\},$$

where $p_{L,k}$, $p_{U,k}$ are constant vectors. For the sake of notation, let us denote by $\text{p}\in R^{n_{p}}$ the vectors of unknown parameters to be estimated, with $n_{p}=n_{\text{par}}n_{\text{ph}}$, and let Y=Y(t,p) highlight the dependence of the states on the parameters. Consequently, $P_{ad}$ is the $n_{p}$-dimensional hypercube delimited by the constraints (3.3). Additional constraints on the parameters are assumed, by imposing that some of them are constant over all phases.

Let Δt be a positive time step, for which we consider $n_{\text{me}}$ measurements of $n_{\text{com}}=5<n_{e}$ compartments at equally spaced times $t_{j}=j\Delta t$, $j=1,\ldots ,n_{\text{me}}$ over the interval $I=[t_{I},t_{F}]$, with $t_{1}=t_{I}+\Delta t$, $t_{n_{\text{me}}}=t_{F}$; in total, we have $n_{\text{com}}\times n_{\text{me}}=5\times n_{\text{me}}$ reported data, say $\hat{D}(t)={\left\{{\hat{\text{Y}}}_{I,H,T,E,R_{D}}(t_{j})\right\}}_{j=1}^{n_{\text{me}}}\in R^{5\times n_{me}}$, that is,

3.4 $$\begin{array}{r} \hat{D}(t)=\{{\left(\hat{I}(t_{1}),\hat{H}(t_{1}),\hat{T}(t_{1}),\hat{E}(t_{1}),{\hat{R}}_{D}(t_{1})\right)}^{T},\ldots ,(\hat{I}(t_{n_{me}}),\hat{H}(t_{n_{me}})),\hat{T}(t_{n_{me}}),\hat{E}(t_{n_{me}}),{\hat{R}}_{D}{\left(t_{n_{me}}\right)}^{T}\}. \end{array}$$

The first stage of the calibration process is then performed by seeking a LS estimate of the parameters vector, given by the solution of the following minimization problem:

3.5 $$\hat{\text{p}}=\underset{\text{p}\in P_{ad}}{\text{arg min}}J(\text{p}),$$

with

3.6 $$J(\text{p}):=\sum_{j=1}^{n_{me}}\sum_{k=\{I,H,T,E,R_{D}\}}\alpha_{k}(t_{j})||{\text{Y}}_{k}(t_{j},\text{p})-{\hat{\text{Y}}}_{k}(t_{j})|{|}_{2}^{2},$$

where ${\text{Y}}_{k}(t_{j},\text{p})$, $k=\{I,H,T,E,R_{D}\}$ denote the components of solution Y of problems (3.1) and (3.2), corresponding to the compartments I,H,T,E and $R_{D}$, respectively, evaluated at time instant $t_{j},j=1,\ldots ,n_{\text{me}}$, for the set of parameters p. For a balanced distribution of the error across the different compartments, whose amplitudes vary with time, the dynamical weight coefficients are defined as $\alpha_{k}(t_{j})=1/{\hat{\text{Y}}}_{k}(t_{j})$.

We considered the official epidemiological data supplied daily by the Dipartimento della Protezione Civile (Italian Civil Protection Department), hereafter called ‘raw data’ and which are freely available at https://github.com/pcm-dpc/COVID-19 [17]. The accuracy of these data is highly questioned, in particular concerning the estimate of the total number of reported positive cases (strongly dependent on the daily screening effort) [4]. This lack of accuracy is indeed one of the most critical aspects of simple SIR-like models in which the calibration of the infected compartment is often performed using data on the reported positive cases. In our model, the $n_{\text{com}}=5$ time series selected for the calibration (*isolated, hospitalized, threatened, extincts* and *recovered from detected*) are in fact the data that have been supplied daily by the Italian authorities since the beginning of the pandemic. One of the key features of the proposed SUIHTER model is indeed the one-to-one correspondence of the compartments with the categories for which reliable data, such as those provided on a daily basis by the Italian Civil Protection Department, are available [17].

When $n_{\text{ph}}$ phases are considered, equation (3.5) leads to the optimization of $n_{p}=14n_{\text{ph}}$ parameters in total. Namely, for each phase of the epidemic, we have the 14 parameters given by $\left[\beta_{U},\beta_{I},\beta_{H},\omega_{I},\omega_{H},\delta ,\rho_{U},\rho_{I},\rho_{H},\theta_{H},\theta_{T},\gamma_{I},\gamma_{H},\gamma_{T}\right]$.

Unfortunately, so many parameters make the calibration process problematic. In what follows, we calibrate our model under the following simplifying assumptions:

— $\beta_{I}$ and $\beta_{H}$ are set to zero, by assuming that the infection only occurs through a contact between a susceptible individual and an undetected infected individual (see, for instance, [29]);
— $\theta_{H}$ is set to zero as we assume that a hospitalized individual can return home only once that individual has recovered, since this parameter may be difficult to estimate in the absence of specific data on the H to I flux;
— $\gamma_{H}$ are set to zero, by assuming that, when a hospitalized individual is in a life-threatening condition, that individual is moved to ICU;
— δ, $\rho_{U}$, $\rho_{I}$, $\rho_{H}$, $\gamma_{I}$, $\theta_{T}\in R$ are constant on $\left[t_{I},t_{F}\right]$.

With these restrictions, the total number of parameters to be calibrated is reduced to $4n_{ph}+6$.

The first stage of the calibration process has been performed by solving the minimization problem (3.5) numerically. We have used a parallel version of the limited memory Broyden–Fletcher–Goldfarb–Shanno algorithm with box constraints (L-BFGS-B); see [30] for details.

The second stage of the calibration process aims to quantify uncertainties and has been carried out by employing a Bayesian framework, since the latter provides probability densities of the input parameters that can be propagated through the model.

Bayesian inference allows us to construct a probability distribution function (PDF) for the unknown parameters, merging prior information and available data, the latter entering in the expression of the likelihood function. At this stage, in order to account for the uncertainty on the initial conditions, we extend the set of parameters to be estimated to $\bar{\text{p}}=(\text{p},\text{q})$, where $\text{q}=(U(t_{I}),R(t_{I}))$ collects the initial conditions for the *undetected* and *recovered* compartments whose values are not available from the data and can only be estimated. The posterior PDF can then be obtained through the Bayes theorem on conditional probabilities. For the case at hand, we quantify the likelihood of the parameter vector $\bar{\text{p}}$ corresponding to the model outcome ${\text{Y}}_{k}(t_{j},\bar{\text{p}})$, $k=\{I,H,T,E,R_{D}\}$ in correlation with the reported cases $\hat{D}(t)$ as

$$\pi (\hat{D}(t)|\bar{\text{p}})=\prod_{k=\{I,H,T,E,R_{D}\}}\frac{1}{{\left(2\pi \sigma^{2}\right)}^{n_{me}/2}}\exp\left(-\frac{1}{2\sigma^{2}}\sum_{j=1}^{n_{me}}{\left({\hat{\text{Y}}}_{k}(t_{j})-{\text{Y}}_{k}(t_{j},\bar{\text{p}})\right)}^{2}\right),$$

where the (unknown) variance $\sigma^{2}$ is assumed to be constant for each compartment.

Note that a usual assumption when estimating parameters of epidemiological models is that reported cases, given their discrete nature, follow either a Poisson or a negative binomial distribution [4,23,28,29]. However, given the relatively large number of cases, the normal distribution represents a flexible approximation, allowing for faster computations.

Using Bayes’ theorem, we obtain the posterior distribution of the parameters $\bar{\text{p}}$ accounting for the prior knowledge on the parameters and the reported cases, as

$$\pi (\bar{\text{p}}|\hat{D}(t))=\frac{\pi (\hat{D}(t)|\bar{\text{p}})\pi (\bar{\text{p}})}{\pi (\hat{D}(t))}=\frac{\pi (\hat{D}(t)|\bar{\text{p}})\pi (\bar{\text{p}})}{\int_{P}\pi (\hat{D}(t)|\bar{\text{p}})\pi (\bar{\text{p}})\;\text{d}\bar{\text{p}}},$$

where $\pi (\bar{\text{p}})$ denotes the (uniform) prior distribution for the parameters. Here, we assume that the prior PDF for the model parameters p is centred at the LS estimate $\hat{\text{p}}$ obtained during the former calibration stage, on a range $\left[0.9\hat{\text{p}},1.1\hat{\text{p}}\right]$, while the prior of the initial values q is centred around an estimate $\hat{\text{q}}$ obtained based on the IFR (see equation (2.3)), on a range $\left[0.7\hat{\text{q}},1.3\hat{\text{q}}\right]$. The larger relative amplitude of the latter prior interval reflects the higher uncertainty on the initial value for the *recovered* and *undetected* compartments.

An alternative, more common and rigorous procedure would require informative priors to be specified for the parameters, starting from key epidemiological features, as done, for example, in [4,29]. However, given the large numbers of parameters to be estimated—some of which do not find explicit counterparts in the epidemiological literature—we have assumed uniform priors, centred about the LS estimates, as a practical shortcut to overcoming the difficulty in specifying the prior distribution. In terms of predictive capability of the model, the numerical results provided in §4 allow us to assess the proposed approach.

Since we cannot obtain the posterior distribution over the model parameters p analytically, we adopt approximate-inference techniques based on Monte Carlo (MC) methods, which aim to generate a sequence of random samples from a Markov chain whose distribution approaches the posterior distribution asymptotically, whence the name of MCMC [31]. In particular, we have used the delayed rejection adaptive Metropolis (DRAM) algorithm [32] implemented in pymcmcstat; see [33] for the details. The first 500 000 samples of the chain serve to tune the sampler and are later discarded (burn-in period). We use the next 500 000 samples to approximate the posterior distribution for the parameters $\bar{\text{p}}$.

From the generated chains, we draw $N_{\text{MC}}$ samples of the parameters ${\bar{p}}_{1},\ldots ,{\bar{p}}_{N_{\text{MC}}}$ that we use to perform forward propagation of uncertainty through the model, and to compute predictive envelopes of the SUIHTER model compartments (or predictive distributions).

We report the MC samples of the trajectories on the time interval $\left(t_{I},t_{\text{for}}\right]$, including a forecast window $\left(t_{F},t_{\text{for}}\right]$ that extends beyond the time window $\left(t_{I},t_{F}\right]$ where data have been reported, to assess the predictive capability of the model.

## 4. Results and discussion

In this section, we present three batteries of numerical results assessing the forecasting capabilities of the SUIHTER model. Our analysis focuses on the second wave of the epidemic that started at the end of summer 2020 and, at the time of writing, is still affecting Italy. In §4a, we present the simulation of the second wave obtained with the SUIHTER model using for its calibration all the data between 20 August 2020 and 31 December 2020. By limiting the time range of the data used for the calibration, we also investigate the model's capability of forecasting the peaks of the different compartments (see §4b).

Our results at the national level for the second outbreak have been obtained by initializing the *isolated*, *hospitalized*, *threatened* and *extinct* compartments with the data provided by the Italian Civil Protection Department [17] on 20 August 2020, namely I=15 063, H=883, T=68 and E=35 418. The initial values for the *undetected* and *recovered* compartments are estimated using the strategy based on IFR and the time-dependent CFR introduced in §2a, resulting in the values U=12 274 and R=2 916 082, respectively. Finally, the initial condition for the *susceptible* compartments is given by S=(N−I−U−H−T−E−R)=57 504 185. Note that this would imply that, by the end of the first wave, around 4.8% of the Italian population had been infected. A serosurvey organized by Istituto Nazionale di Statistica (ISTAT) and Istituto Superiore di Sanità (ISS) had estimated that 2.5% of the Italian population had been infected [34,35]; the survey however had a low compliance, so that its results may be biased. A corresponding survey in Spain [36] with a much higher compliance rate estimated a seropositivity value of 4.6% or 5%, depending on the methodology used for the seroprevalence analysis. Using an ensemble model calibrated over several countries [37] the estimate of the proportion infected in Italy on 1 September 2020 was around 4.5%. Using instead a dynamical model calibrated over detailed data [28] the estimate of the proportion infected in Italy on 30 September 2020 was 4.78%. Thus, the value of *recovered* cases obtained for 20 August looks rather realistic.

### (a) Simulation of the second epidemic wave

The SUIHTER model has been used to simulate the second epidemic outbreak, from 20 August 2020 until 31 December 2020. The different phases in which the parameters can take different values have been identified according to the occurrence of some criticalevents:

— 24 September 2020: all schools at the national level reopened after the summer (and spring lockdown) closure (schools calendars vary by grades and by region in Italy);
— 8 October 2020: new rules imposing the mandatory use of masks in all locations (either indoor or outdoor) accessible to public;
— 26 October 2020: confinement rules including distance learning for most secondary schools, limitations on the activity of shops, bars and restaurants, strong limitation on sport and leisure activities;^1^
— 6 November 2020: stricter confinement rules including distance learning from the 9th grade, further restrictions on commercial activities, limitations on circulation outside people's own municipality (for some Italian regions, classified as *red* regions);^2^
— 15 November 2020: additional confinement rules as more regions became *red* regions;^3^
— 19 November 2020: additional confinement rules as more regions became *red* regions;^4^
— 29 November 2020: relaxation of confinement rules in some regions as they became *orange* regions;^5^
— 6 December 2020: relaxation of confinement rules in some regions as they became *yellow* regions;^6^
— 18 December 2020: stricter confinement rules are introduced for the Christmas holidays.^7^

By considering a time lag of 4 days (to account for the incubation period) [38], the corresponding phases on which the model parameters are defined (and possibly changing) are:

— phase 1: 20 August 2020–28 September 2020;
— phase 2: 29 September 2020–11 October 2020;
— phase 3: 12 October 2020–29 October 2020;
— phase 4: 30 October 2020–9 November 2020;
— phase 5: 10 November 2020–18 November 2020;
— phase 6: 19 November 2020–23 November 2020;
— phase 7: 24 November 2020–3 December 2020;
— phase 8: 4 December 2020–10 December 2020;
— phase 9: 11 December 2020–22 December 2020;
— phase 10: 23 December 2020–31 December 2020.

As mentioned in §3, the compartments employed for calibration are only those with more reliable data, namely *isolated* (*I*), *hospitalized* (*H*), *threatened* (*T*), *extinct* (*E*) and *recovered from detected* individuals.

We performed the model calibration by employing the MCMC parameter estimation procedure described in §3, over the 10 phases, using the data over the full time range from 20 August 2020 to 31 December 2020. The simulations were run for the subsequent 15 days beyond the date associated with the last set of data used for the calibration forecasting the evolution of the epidemic until 15 January 2021. For the new additional phase, the values of the parameters are obtained by linearly extrapolating the two (constant) values of the corresponding parameter of the last two phases, located on the final day of each phase, namely phases 9 and 10.

**Figure 2.**
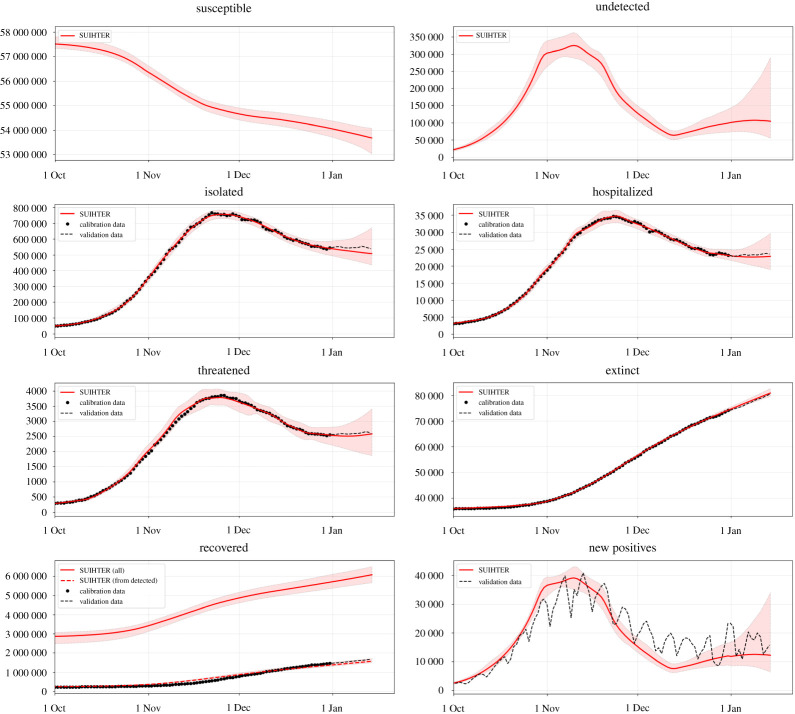
Expected values (solid lines) and 95% prediction intervals (shaded areas) for the seven compartments of the SUIHTER model plus the additional *daily new positives* compartment. The data are indicated with black dots (in the calibration phase) and with a dashed line in the validation phase. (Online version in colour.)

In figure 2, we report the expected values for the time evolution of the seven compartments of the SUIHTER model as well as the time evolution of the additional compartment of the *daily new positives*, which corresponds to δ U(t), and the corresponding 95% prediction intervals obtained by propagating input uncertainties through the model.

We note that the calibrated compartments (*isolated*, *hospitalized*, *threatened*, *extinct* and *recovered from detected*) accurately fit the corresponding time series-data in the calibration phase. For all compartments the 15-day forecast also indicates the capability of the model in predicting the evolution of the epidemic at the national level.

Moreover, the time history of the *daily new positives* is also in reasonable agreement with the data, proving that the model is also able to capture the main dynamics of the system for quantities that are not directly driven by the data calibration. Our calibration indicates that, from 20 August 2020 to 15 January 2021, 4 056 118±284 533 individuals were infected, of which 48.0%±2.0% had been detected. In addition, we estimate *a posteriori*, i.e. by using the outputs of the simulation, that the IFR during the same period was 1.3%±0.1%. This latter figure is compatible with the IFR estimated at 1.2% for Italy by using estimates by age reported in [19], but is somewhat higher than the estimate shown in [37]. We also observe that our calculated estimates are likely to be underestimated as the second outbreak is still ongoing at the present time and compartments of isolated and extinct individuals become populated at different time scales.

The median values and the 95% credibility intervals computed by the MCMC calibration are reported in table 1 for the parameters that are constant over the simulation and for the initial values of *undetected* and *recovered*, while in table 2 we report the parameters that are free to change in each phase. The posterior distributions for all the constant and time-dependent parameters are reported in electronic supplementary material, figures S1 and S2. The traceplots for all the parameters (also available in electronic supplementary material, figures S3 and S4) indicate that the MCMC method showed good convergence after the first 500 000 samples.

**Table 1.** Median values and 95% credibility intervals (CI) of constant parameters and U and R initial values.

	median	95% CI
δ	0.12041	[0.10739, 0.12841]
$\gamma_{I}$	$3.78\times 10^{-5}$	$\left[3.43\times 10^{-5},\;4.15\times 10^{-5}\right]$
$\rho_{U}$	0.12320	[0.11303, 0.13593]
$\rho_{I}$	0.02408	[0.02197, 0.02658]
$\rho_{H}$	0.06677	[0.06171, 0.07212]
$\theta_{T}$	0.05026	[0.04517, 0.05456]
$U(t_{I})$	12 571	[9346, 15 775]
$R(t_{I})$	2 551 280	[2 270 830, 2 832 576]

**Table 2.** Median values and 95% credibility intervals (CI) of the parameters that change over the phases and the corresponding$R_{0}$.

	$\beta_{U}$		$\omega_{I}$	
phase	median	95% CI	median	95% CI
1	0.2640	[0.2475, 0.2825]	0.0059	[0.00537, 0.00648]
2	0.3658	[0.3329, 0.3936]	0.00771	[0.00701, 0.00847]
3	0.3449	[0.3223, 0.3685]	0.00933	[0.00849, 0.01018]
4	0.2756	[0.2485, 0.2972]	0.00691	[0.00629, 0.00755]
5	0.2421	[0.2202, 0.2658]	0.00496	[0.00445, 0.00537]
6	0.1779	[0.1615, 0.1952]	0.00422	[0.00383, 0.00464]
7	0.2093	[0.1906, 0.2307]	0.00340	[0.00309, 0.00373]
8	0.1924	[0.1743, 0.2109]	0.00313	[0.00283, 0.00342]
9	0.3052	[0.2780, 0.3354]	0.00309	[0.00281, 0.00339]
10	0.2949	[0.2686, 0.3251]	0.00351	[0.00319, 0.00385]
	$\omega_{H}$		$\gamma_{T}$	
phase	median	95% CI	median	95% CI
1	0.0132	[0.0121, 0.0146]	0.0760	[0.0691, 0.0837]
2	0.0192	[0.0173, 0.0210]	0.1252	[0.1133, 0.1372]
3	0.0223	[0.0202, 0.0243]	0.0886	[0.0793, 0.0958]
4	0.0264	[0.0238, 0.0286]	0.1561	[0.1400, 0.1689]
5	0.0259	[0.0233, 0.0281]	0.1673	[0.1517, 0.1830]
6	0.0269	[0.0243, 0.0293]	0.1909	[0.1741, 0.2103]
7	0.0263	[0.0238, 0.0286]	0.1900	[0.1726, 0.2079]
8	0.0251	[0.0226, 0.0272]	0.1872	[0.1708, 0.2055]
9	0.0244	[0.0223, 0.0269]	0.1924	[0.1729, 0.2086]
10	0.0249	[0.0226, 0.0272]	0.1867	[0.1700, 0.2053]

The former parameters and time-dependent functions represent rates that can be used to interpret the dynamics of the second Italian outbreak. For example, large values of $\beta_{U}$ indicate sustained transmission rates at the corresponding phases. Values of healing rates $\rho_{U}$, $\rho_{I}$ and $\rho_{H}$ are proportional to the probability of healing for individuals in the compartments *U*, *I* and *H*, but are inversely proportional to the corresponding average time of healing; the rate $\rho_{I}$ also incorporates the healing on isolated individuals who are however asymptomatic. To better understand the role of the parameters, note that, if they were constant, $\rho_{I}/(\rho_{I}+\omega_{I}+\gamma_{I})$ would represent the probability of an isolated individual recovering without being hospitalized, and similarly $\rho_{H}/(\rho_{H}+\omega_{H}+\gamma_{H})$ represents the probability of a hospitalized individual recovering without being transferred to an ICU. In the same way, $\gamma_{T}/(\gamma_{T}+\theta_{T})$ represents the probability of an individual in an ICU dying, and $\delta /(\delta +\rho_{U})$ represents the probability that an infected individual is detected.

Finally, table 2 also reports the value of the basic reproduction number $R_{0}$ calculated as in equation (2.2) for the SUIHTER model. The calculation uses the model parameters reported in tables 1 and 2 (columns 1--4). Note that the amplitude of the credibility intervals is strongly influenced by the choice of the prior in the interval centred about the values of the model parameters obtained by the LS procedure ±10%. They should mainly be judged in relative terms.

We observe that the value of $R_{0}$ obtained by the calibration reflects the full reopening of educational activities and work restaring after holidays, as well as the public health measures and restrictions later introduced by the authorities to contain the second epidemic outbreak. In particular, the rise of $R_{0}$ in phases 2 and 3 follows the full reopening of schools and restarting of working activities from mid-September, and probably accounts for seasonality effects too. Restrictions on mobility, schools and businesses and partial lockdowns were introduced in late October at the regional and national levels, as reflected by the decrease in $R_{0}$ from phase 5 to phase 6, when $R_{0}$ became less than 1. Partial reopening and easing of restrictions were gradually introduced in some regions and at the national level from late November, as the new increment of $R_{0}$ from phase 9 indicates.

#### (i) Simulating the second outbreak for Italian regions

The results obtained by simulating the epidemic at the national scale can indeed hide specific local outbreaks. The SUIHTER model can also simulate the evolution of the epidemic for everyone in the 20 Italian regions for which the same time-series data as those used for the national calibration are available. Unfortunately, this is not true for the finer geographical level (the 107 provinces) since only the number of total cases from the beginning of the epidemic is provided.

**Figure 3.**
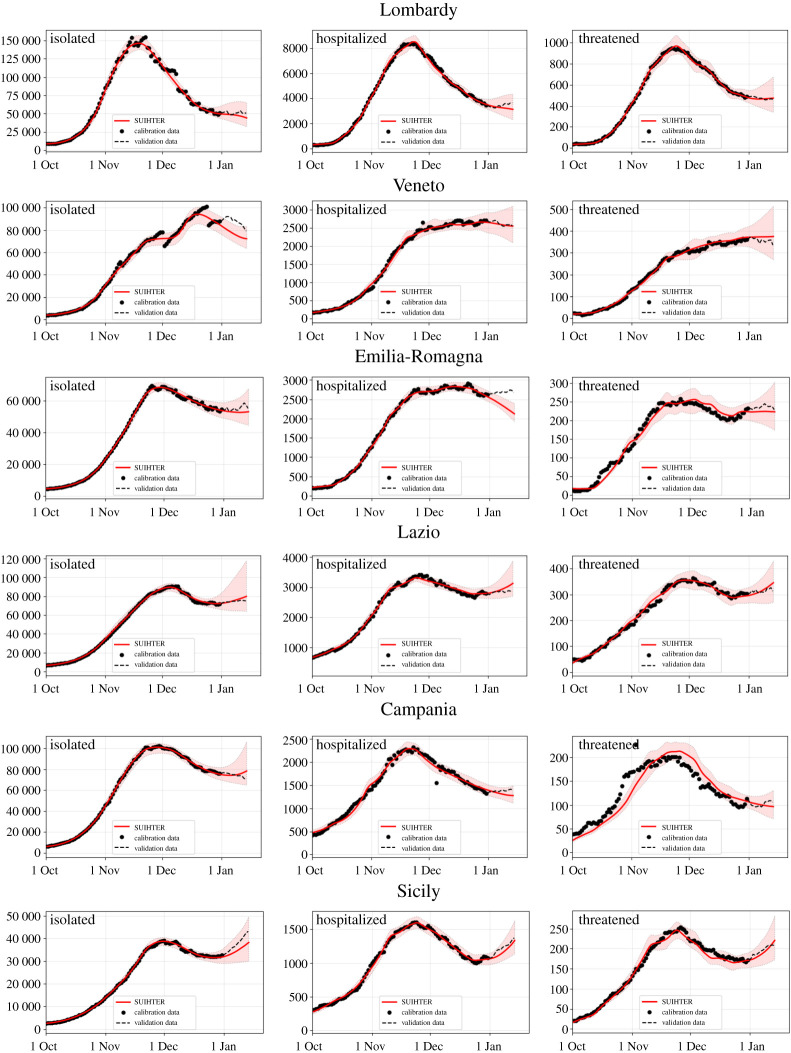
Expected values (solid lines) and 95% prediction intervals (shaded areas) for the *isolated*, *hospitalized* and *threatened* compartments, from left to right, in the six larger Italian regions. (Online version in colour.)

Following the same initialization and calibration strategies adopted at the national level, we have carried out the simulation of the second epidemic outbreak in the six larger Italian regions, namely Lombardy, Veneto, Emilia-Romagna, Lazio, Campania and Sicily. In figure 3, the expected value for the time evolution of the three infectious compartments used for the calibration and the corresponding 95% prediction intervals are reported for the former six regions. The calibration has been carried out using the same setting as for the national level, i.e. calibrating the model with the data available until 31 December 2020 and then simulating until 15 January 2021. The results obtained by numerical simulations are in good agreement with the real data, with few exceptions, namely the *isolated* compartment in Veneto (where the time series is clearly affected by some reporting problems) and the *hospitalized* compartment in Emilia-Romagna.

### (b) Predicting the peaks

Predicting the peak of an epidemic outbreak is a tremendous challenge for an epidemiological model. Yet, the predictive capability of epidemiological models is of paramount importance to inform policymakers about the dynamics of the disease and foresee the timing and levels of the peaks of infected, hospitalized and ICU-treated individuals, as well as the potential effects of policy responses.

With the goal of investigating to which extent our SUIHTER model is able to predict the occurrence of the epidemic wave peak, we repeated the calibration using the data over limited time ranges.

In particular, we have considered three different cases: in *case 0* we used all the data time histories available until 3 December, while in *cases 1*, *2* and *3*, the data employed for the calibration were limited to 23 November, 18 November and 9 November, respectively. For each case, the simulations were run for the subsequent 30 days beyond the date associated with the last set of data used for the calibration and the linear extrapolation carried out as indicated before.

**Figure 4.**
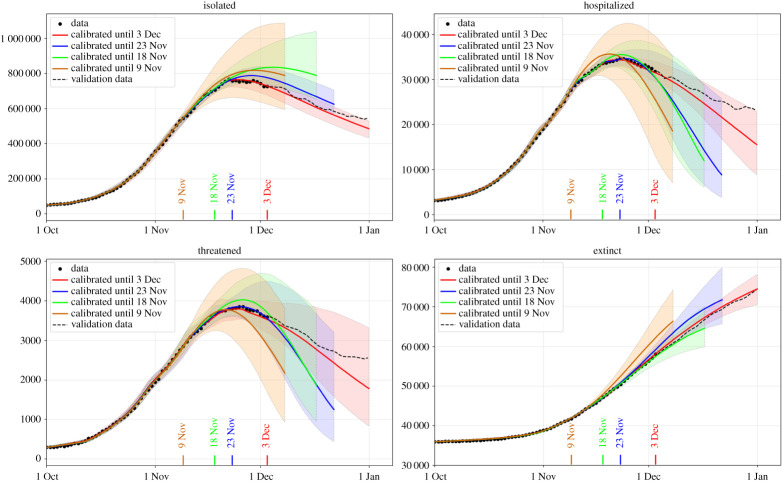
Peak forecast obtained by the SUIHTER model with different data ranges for the *isolated*, *hospitalized*, *threatened* and *extinct* compartments. (Online version in colour.)

In figure 4, we report the expected value for the time evolution of the four compartments used for the calibration, and the 95% prediction intervals obtained by propagating input uncertainties through the model. The accuracy of the forecast, as expected, improves when a richer set of data are employed in the calibration. Our simulations show the occurrence of a peak for each of the three compartments, not only for case 0, in which the time lapse of the data used for the calibration covers the peaks, but also for cases 1, 2 and 3, when the time-series data employed for the calibration are still rising. However, we should remark that if the model is calibrated with a shorter time series, namely available data stop more than 30 days before the peak, the occurrence of the peak cannot be correctly predicted.

As already noticed, because of the overall complexity of the problem and the limited data available for its calibration, we do not intend here to certify in rigorous terms the actual values of the future compartments. However, in spite of the widths of the predictive intervals (which depend, to some extent, on the widths of the chosen prior distributions), we nonetheless observe that the expected values (solid lines in figure 4) carry meaningful prediction capabilities.

To further assess the accuracy of the prediction, it is interesting to compare this peak forecasting with respect to the actual data, i.e. the day and value that have been reported for the different compartments at the end of November 2020. Moreover, we propose a comparison with the predictions obtained using the two different strategies based on data fitting. The first is based on a simple polynomial fit of degree 2 on the last recorded 10 days, while the second is obtained by using a curve registration (see [39] for an overview on this subject) by exploiting the similarities between the first and second waves. The registration procedure is performed by first computing the exponentially modified Gaussian (EMG) function that best fits the first wave. We denote this function as w(t), $t_{0}\leq t\leq t_{1}$, with $t_{0}$ the first day of the recorded data (24 February) and $t_{1}$ equal to 1 August. Then a second minimization problem is solved to compute the time shift and scaling factors to apply to the computed EMG function to best fit the rising portion of the second wave in the time range $\left[t_{k},t_{n}\right]$, with $t_{k}$ coinciding with 15 October and $t_{n}$ with the last recorder date. Namely, we look for the optimal time shift $\bar{h}$ and the scaling factors ${\bar{s}}_{1}$ and ${\bar{s}}_{2}$ such that

$$(\bar{h},{\bar{s}}_{1},{\bar{s}}_{2})=\underset{h,s_{1},s_{2}}{\text{arg min}}\sum_{i=t_{k}}^{t_{n}}(s_{1}\;w{\left(s_{2}\;t_{i}+h)-d_{i}\right)}^{2},$$

where $d_{i}$ is the value of the considered data series at day $t_{i}$.

For each data series, the fitted EMG function and the optimal values for the shift and scaling factors are computed and, in this way, the shape of the first wave can be used to complete the second wave for the different compartments.

**Figure 5.**
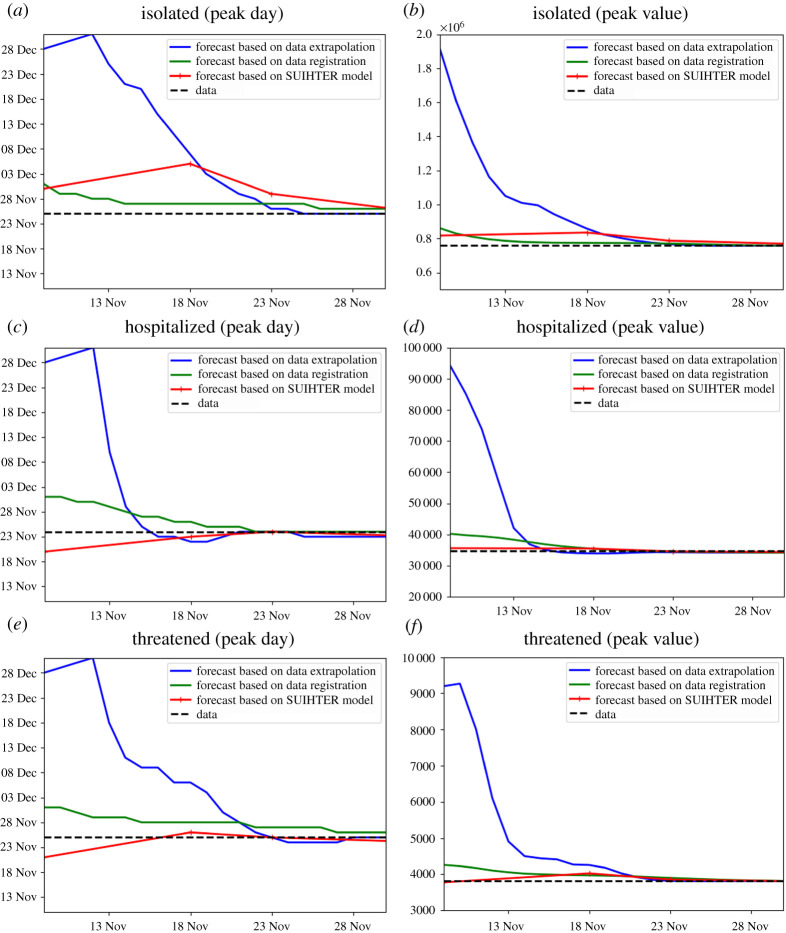
Peak day (*a*,*c*,*e*) and peak value (*b*,*d*,*f*) versus last used data by day for the three compartments *isolated* (*a*,*b*), *hospitalized* (*c*,*d*) and *threatened* (*e*,*f*), estimated with data extrapolation, data registration and the SUIHTER model. (Online version in colour.)

A comparison between the peak forecast obtained with the SUIHTER model, the quadratic extrapolation (based on the last 10 days) and the registration approach is displayed in figure 5, for the *isolated*, *hospitalized* and *threatened* compartments. The curves show how the prediction in terms of the day of peak occurrence and peak value changes when an increasing number of data are used (the last data day is reported on the horizontal axis). To minimize the effect of daily data noise, the reference value (dashed line) is obtained by smoothing the data with a Savitzky–Golay polynomial smoothing filter of degree 3 [40].

By comparing the peak predictions with the day and value of the measured (smoothed) data peak (shown with a dashed line in figure 5), we should first remark that the SUIHTER prediction largely outperforms those obtained with polynomial extrapolation. Moreover, even when compared with predictions based on the registration with the first epidemic wave, the SUIHTER model is more accurate for most of the considered quantities. When making this comparison, it is worth noticing that, while prediction based on the registration strongly depends on the evolution of the different compartments during the first epidemic wave, the predictions based on SUIHTER do not require any *a priori* knowledge of previous epidemic waves.

## 5. Conclusion and model limitations

In this paper, we have introduced a new mathematical model, named SUIHTER, to describe the ongoing pandemic of COVID-19. This epidemiological model is constructed on seven compartments—susceptible uninfected individuals (S), undetected (both asymptomatic and symptomatic) infected (U), isolated (I), hospitalized (H), threatened (T), extinct (E) and recovered (R)—and we exploit it to study and analyse the second Italian outbreak that emerged in autumn 2020 and is still ongoing. In particular, our model is suited for calibration against data made available daily by the Italian Civil Protection Department [17]. On the basis of these data at the national level, our calibration populates the compartments I, H, T, E and $R_{D}$, which we purposely use to determine transmission rates, rates of recovery, IFR, etc. In particular, SUIHTER allows us to estimate the infected but undetected population, a compartment (U) that is crucial for studying and understanding the epidemic, especially considering that large numbers of infected individuals went uncounted during the first and even the second outbreaks in Italy. Moreover, thanks to our approach, transmission rates, and thus the basic reproduction number $R_{0}$, can be estimated. Finally, our calibration is made robust by exploiting Bayesian estimation using the MCMC method.

The SUIHTER model calibrated at the Italian national level is validated against data related to the last part of the second outbreak. Comparisons are made against basic statistical models, namely quadratic regression and registration of the first epidemic wave. The comparison demonstrates the better accuracy of SUIHTER for predictive purposes. This is made possible by using extrapolated transmission rates that are calibrated at earlier times through regression models, a feature that allows the peaks of the second Italian outbreak to be captured correctly and enables SUIHTER to be used in a predictive fashion by leveraging data available at the current date. This novel approach attempts to circumvent a common issue of the use of epidemiological compartmental models for forecasting [7], that is, accurately capturing transmission rates. However, as our approach is based on interpolating values of these transitions rates, the accuracy of their extrapolation and, consequently, their exploitation for prediction within SUIHTER can only be limited to restricted time windows, especially when government interventions and citizen behaviours are changing. Note that, although the calibration procedure did not make any assumptions about the temporal changes in parameters, the estimates accurately reflect the policy changes: estimates of $R_{0}$ decrease as control measures are tightened and increase when they are relaxed. The results of the simulation of the second wave carried out at the national and regional levels showed the capability of the model in predicting the time evolution accurately in a time frame of 15 days past the data used in the calibration. In longer term predictions the model should account for the possible changes in restriction rules that may occur in the future to supply analyses of different scenarios (as recently done in [40] based on the SUIHTER model).

A further limitation of our approach is that we are currently calibrating the Italian epidemic outbreaks at the national level, that is, as a whole, without summing up the different contributions at the level of the 20 Italian regions for which data are available [17]; however, we performed the calibration of the six larger Italian regions. Populating compartments at the national level by summing up results obtained by tailored calibrations of each Italian region would allow the spatio-temporal heterogeneity of the Italian outbreaks to be better captured, reflecting different mobility patterns and densities of population. In this respect, several different approaches have been proposed in the literature (e.g. [41] and the references therein), ranging from the use of network-based models [42,43] to systems of ODEs on a network [44,45], as well as non-local partial differential equations [46]. Among the contributions that appeared during the COVID-19 pandemic, we also recall [2,4,13], where a meta-community SEIR-like model was proposed and employed to reproduce the contagion in Italy. However, calibrating our SUIHTER model at the regional level, and for all the regions, would require a more sophisticated design owing to the intrinsic ill-posedness of the inverse problem, especially when taking mobility patterns into account. Nevertheless, we plan to better address spatio-temporal heterogeneity of the Italian outbreaks in the future by generalizing our SUIHTER model to incorporate suitable spatial–multi-city mobility terms at the regional level. Even though a more spatially detailed compartment model is desirable, to act, for example, at the provincial level (Italy comprises 107 provinces), currently no detailed data for its calibration have been made available.

Although the SUIHTER model is very sophisticated and involves 14 time-dependent parameters and functions to be determined based on available data, we limited our calibration to a subset of the possible control variables, by forcibly setting to zero some parameters that we deemed to be less relevant for the transmission of the epidemic and by assuming some others as constants over time. We also neglected incubation time, and we implicitly assumed that all distributions in the states are exponential, which is far from correct [47]. Still, we believe that this qualifies as an acceptable compromise among the complexity of the SUIHTER model and its calibration procedure, the associated computational costs and the accuracy of the results. Some of the calibrated parameters assume values that are able to compensate for those parameters prescribed *a priori*, even if their interpretation may not be straightforward in explaining the outbreak. In this respect, we plan to assess the robustness of our approach by allowing the calibration of additional parameters. Furthermore, our multi-compartment SUIHTER model does not consider stratification of age groups within the compartments. This is an important aspect as some compartments such as H, T and E are mostly populated by the elderly, while the transmission mechanisms widely differ by age and context of infection (workplace, school, family, etc.) [28,48]. We also plan to improve SUIHTER by considering age stratification within its compartments.

Among the limitations of our work is the identifiability of model parameters as SUITHER requires a relatively large set of such parameters to be calibrated. Although we acknowledge its importance, the current study does not present a direct verification of the identification conditions on model parameters nor, in general, an identifiability analysis. Some, albeit indirect, verification of the identifiability properties of the model comes from the very large sets of time series on which calibration has been successfully performed. These are however aspects that we may better address in future studies.

Finally, in consideration of the ongoing emergency situation during the second Italian outbreak, we believe that our SUIHTER model is well suited to being used in a predictive manner to support and motivate public health measures. To the best of our knowledge, apart from [49], wherein a SEIRD model is used at the regional level, SUIHTER stands as one of the first models used to analyse the second Italian COVID-19 outbreak and can readily serve the purpose of predicting the short-term epidemic trends and perform longer term scenario analyses.

## Footnotes

1 Decreto del Presidente del Consiglio dei Ministri (Decree of the President of the Council of Ministers; DPCM) 24 October 2020, http://www.governo.it/sites/new.governo.it/files/DPCM_20201024.pdf.

2 DPCM 4 November 2020, http://www.salute.gov.it/imgs/C_17_notizie_5171_0_file.pdf.

3 http://www.regione.abruzzo.it/system/files/atti-presidenziali/ordinanze/2020/ordinanza-n-102.pdf.

4 http://www.salute.gov.it/imgs/C_17_notizie_5197_0_file.pdf.

5 http://www.salute.gov.it/imgs/C_17_notizie_5197_0_file.pdf.

6 https://www.gazzettaufficiale.it/eli/id/2020/12/05/20A06781/sg.

7 Decree Law 18 December 2020, n. 172, https://www.gazzettaufficiale.it/eli/id/2020/12/18/20G00196/sg.

## Data accessibility

Additional data are given in the electronic supplementary material.
